# Perspectives on zinc-based flow batteries

**DOI:** 10.1016/j.fmre.2024.06.002

**Published:** 2024-06-17

**Authors:** Yuqin Huang, Liping Zhi, Ran Bi, Zhizhang Yuan, Xianfeng Li

**Affiliations:** a Division of Energy Storage, Dalian National Laboratory for Clean Energy, Dalian Institute of Chemical Physics, Chinese Academy of Sciences, Dalian 116023, China; b Comprehensive Energy Research Center, Science and Technology Research Institute, China Three Gorges Corporation, Beijing 101199, China; c University of Chinese Academy of Sciences, Beijing 100049, China

**Keywords:** Energy storage, Zinc-based flow batteries, Demonstration systems, Cell stacks, Material chemistries

## Abstract

Zinc-based flow battery technologies are regarded as a promising solution for distributed energy storage. Nevertheless, their upscaling for practical applications is still confronted with challenges, e.g., dendritic zinc and limited areal capacity in anodes, relatively low power density, and reliability. In this perspective, we first review the development of battery components, cell stacks, and demonstration systems for zinc-based flow battery technologies from the perspectives of both fundamental research and engineering applications. The remaining challenges as well as the perspective for zinc-based flow battery technologies are also briefly discussed. We hope this perspective can help researchers and the community to recognize and understand the status of currently developed zinc-based flow batteries and their limitations as well as advancements in different perspectives, further directing future efforts to enhance their performance effectively.

## 1 Introduction

Demands for renewable energies are growing rapidly as the consumption of global fossil energy brings environmental concerns to mankind. The electricity produced from renewables is volatile and intermittent, which is one of the big obstacles for their widespread applications. Energy storage technology, flow battery technologies in particular, is a safe and effective approach to address this issue [1]. Currently, the flow battery can be divided into traditional flow batteries such as vanadium flow batteries, zinc-based flow batteries, and iron-chromium flow batteries, and new flow battery systems such as organic-based flow batteries, which hold great promise for energy storage applications. Among the above-mentioned flow batteries, the zinc-based flow batteries that leverage the plating-stripping process of the zinc redox couples in the anode are very promising for distributed energy storage because of their attractive features of high safety, high energy density, and low cost [2]. Compared with the energy density of vanadium flow batteries (25∼35 Wh L^-1^) and iron-chromium flow batteries (10∼20 Wh L^-1^), the energy density of zinc-based flow batteries such as zinc-bromine flow batteries (40∼90 Wh L^-1^) and zinc-iodine flow batteries (∼167 Wh L^-1^) is much higher on account of the high solubility of halide-based ions and their high cell voltage. Indeed, not all zinc-based flow batteries have high energy density because of the limited solubility of redox couples in catholyte. In addition to the energy density, the low cost of zinc-based flow batteries and electrolyte cost in particular provides them a very competitive capital cost. Taking the zinc-iron flow battery as an example, a capital cost of $95 per kWh can be achieved based on a 0.1 MW/0.8 MWh system that works at the current density of 100 mA cm^-2^ [3]. Considering the maturity of zinc-based flow batteries, current cost analysis methods or models remain to be improved since the costs of control systems as well as other auxiliary facilities such as pipelines, pumps, and electrolyte tanks are not considered. Most importantly, the feasibility and practicality of a zinc-based flow battery system should be taken into consideration. Overall, benefiting from the above features, the zinc-based flow batteries demonstrate promise for stationary energy storage.

In this perspective, we attempt to provide a comprehensive overview of battery components, cell stacks, and demonstration systems for zinc-based flow batteries. We begin with a summary of the common challenges and the corresponding solution strategies based on the material chemistries (electrolyte, electrode, or membrane) of zinc-based flow batteries. Subsequently, we introduce several representative cell stack operations as well as demonstration systems of zinc-based flow batteries and discuss the reasons why their reliability is low. Finally, we provide some perspectives toward constructing high-reliability, high-performance, and low-cost zinc-based flow battery systems.

## 2 Material chemistries for zinc-based flow batteries

**Fig. 1:**
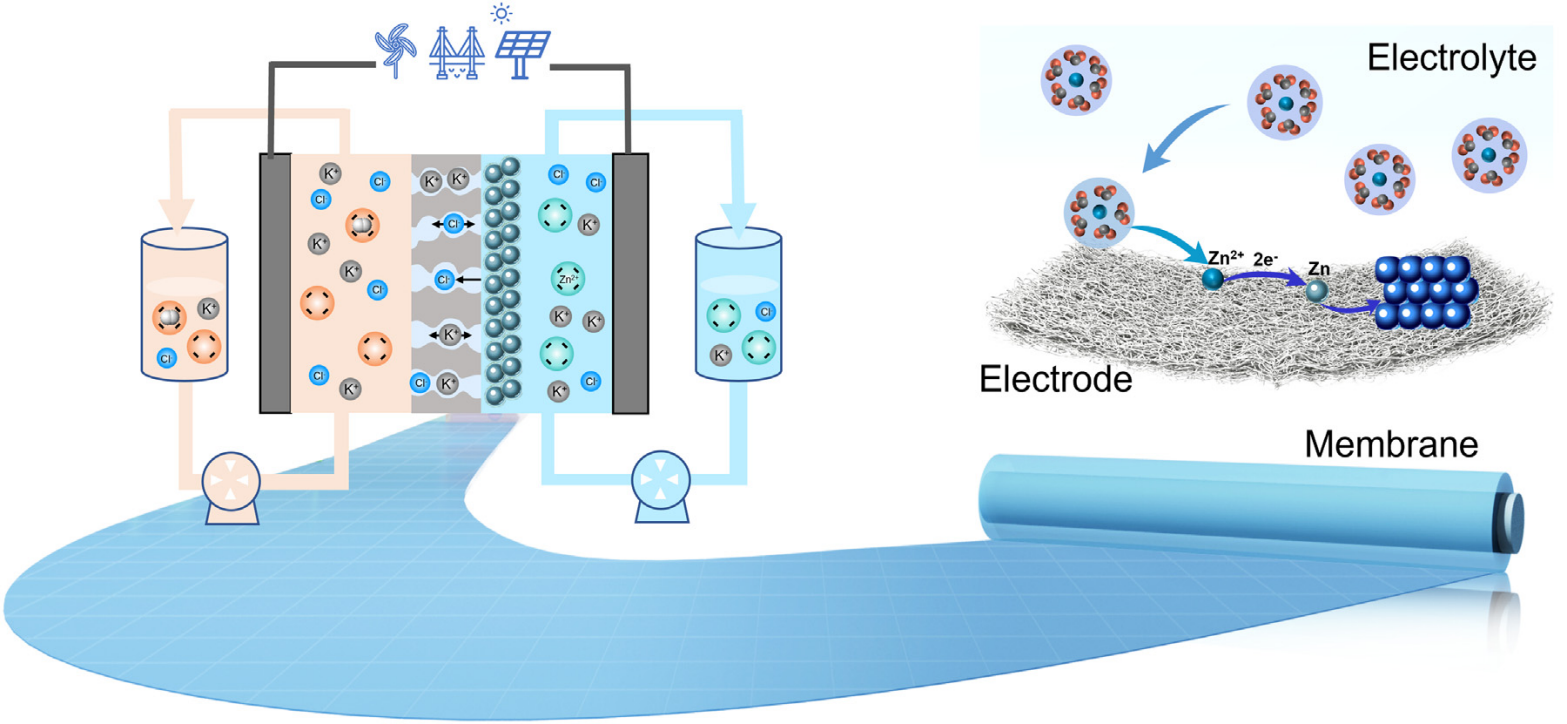
**Schematic illustration of material chemistries in a zinc-based flow battery**.

Since the 1970s, various types of zinc-based flow batteries based on different positive redox couples, e.g., Br^-^/Br_2_, Fe(CN)_6_^4-^/Fe(CN)_6_^3-^ and Ni(OH)_2_/NiOOH [4], have been proposed and developed, with different characteristics, challenges, maturity and prospects. According to the supporting electrolyte used in anolyte, the redox couples in the negative side can be classified into Zn^2+^/Zn (−0.763 V vs SHE) in neutral or acidic media and Zn(OH)_4_^2-^/Zn (−1.22 V vs SHE) in alkaline media [5]. Among the above-mentioned zinc-based flow batteries, the zinc-bromine flow batteries are one of the few batteries in which the anolyte and catholyte are completely consistent. This avoids the cross-contamination of the electrolyte and makes the regeneration of electrolytes simple. Because of the plating-stripping process of zinc species in the anode, the major scientific challenges for all zinc-based flow batteries are common and universal and call for an immediate solution, i.e., achieving a homogenous plating-stripping process of the zinc redox couple on the anode to prevent the formation of dendritic zinc, enhancing the deposited capacity of zinc metal on the anode as well as working current density to decrease the cell stack cost. Normally, the plating of zinc species on the anode experiences three processes: mass transfer, desolvation, and receiving electrons to be reduced to zinc metal [5]. Based on this process, tremendous investigations and efforts are placed on the deposition of zinc species in terms of material chemistries (electrolyte, electrode or membrane). The results have demonstrated that the electrode interfacial engineering to adjust the electrochemical reaction or solvation structure design of zinc species to control their desolvation process is an efficient way to control the plating metallic zinc as well as its morphology (Fig. 1). In addition to the above strategies, innovative membrane design for managing the heat or mass transfer process in membrane-electrode interface is also very effective in addressing the issues of zinc dendrite [6,7]. For instance, taking the advantage of the decoupled design of functional layer and substrate for composite membranes, boron nitride nanosheets (BNNSs) with high thermal conductivity, montmorillonite (MMT) with negatively charged property or carbon nanotubes with high electrical conductivity can be introduced on the surface of the substrate [[6], [7], [8]], which can effectively regulate the surface temperature, zinc species’ concentration and electric field distribution, further enabling the uniform plating of metallic zinc. More importantly, uncovering the ion transport behavior in a membrane favors guiding the membrane structure's design, which enables the zinc-based flow battery to work at a current density as high as 260 mA cm^-2^ (maintaining energy efficiency of 80%) [9,10] and favors advancing the development and application of alternative sustainable membranes for zinc-based flow batteries. Benefiting from the uniform zinc plating and materials optimization, the areal capacity of zinc-based flow batteries has been remarkably improved, e.g., 435 mAh cm^-2^ for a single alkaline zinc-iron flow battery, 240 mAh cm^-2^ for an alkaline zinc-iron flow battery cell stack [11], 240 mAh cm^-2^ for a single zinc-iodine flow battery [12]. Nevertheless, the plating process of the zinc redox couple on the anode makes decoupling for power and energy not suitable for zinc-based flow battery systems. In comparison to the currently reported areal capacity, it suggests that alkaline zinc-based flow batteries can afford a much higher areal capacity than that of neutral or acidic zinc-based flow batteries. This significant difference is mainly derived from the different deposition mechanisms of zinc species in different media, which results in a loose zinc morphology in alkaline media and a dense zinc morphology in neutral or acidic media. In addition to zinc dendrite, electrodeposited metallic zinc is susceptible to corrosion across diverse electrolyte solutions, which can be divided into water corrosion and alkaline corrosion [13]. Water corrosion is the oxidation of the zinc metal surface by water, while alkaline corrosion is the chemical reaction between OH^-^ and zinc metal. Both of them decrease the discharge capacity of the battery and cause active material accumulation in the catholyte. Although the corrosion of zinc metal can be alleviated by using additives to form protective layers on the surface of zinc [14,15], it cannot resolve this issue essentially, which has challenged the practical application of zinc-based flow batteries. In addition to the anode, immense challenges associated with catholyte also remain for practical application of the zinc-based flow batteries, e.g., the relatively low kinetics of complexed bromine redox couple and Ni(OH)_2_/NiOOH couple, the low solubility and stability of ferro/ferricyanide redox couple in strong alkaline conditions at low temperature, and the hydrolysis of Fe^2+^/Fe^3+^ in neutral conditions. Although better electrode or catholyte chemistries have been designed to improve the above issues, most of them are efficient only at laboratory-scale flow battery testing. Their application and demonstration in industrial-scale zinc-based flow battery cell stack is rarely reported, which challenges the batteries’ development.

## 3 Stack operations for zinc-based flow batteries

**Fig. 2:**
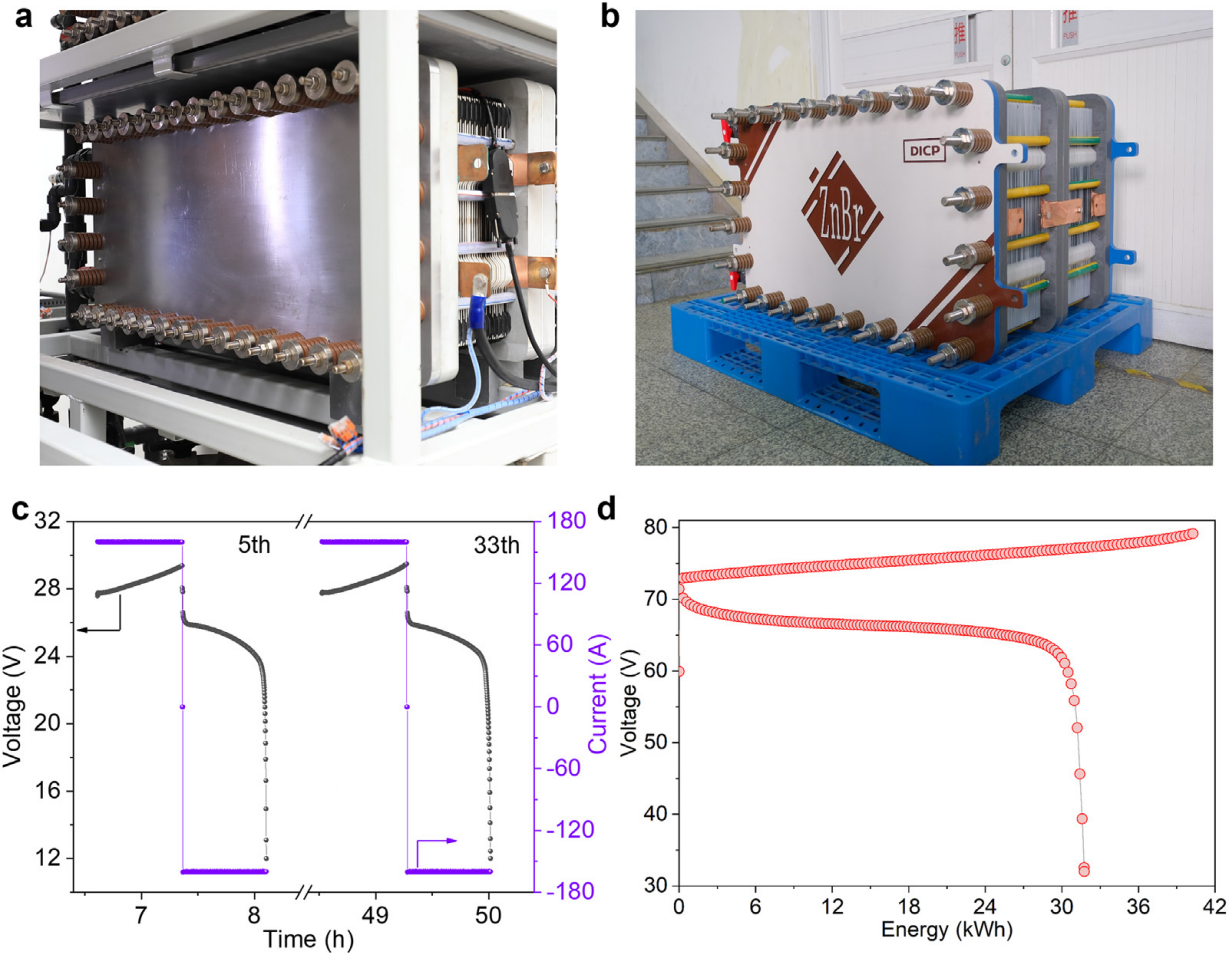
(a) Photograph of 4000 W alkaline zinc-iron flow battery cell stack. Reproduced with permission [11]. Copyright 2022 Elsevier Inc. (b) Photograph of 30 kWh zinc-bromine flow battery cell stack. (c) The voltage (current)-time curves of 4000 W alkaline zinc-iron flow battery cell stack operated at 80 mA cm^-2^. Reproduced with permission [11]. Copyright 2022 Elsevier Inc. (d) The voltage-energy curve of 30 kWh zinc-bromine flow battery cell stack.

To bridge the gap between laboratory-scale development of battery components and industrial-scale zinc-based flow battery stack operation, tremendous research work on cell stack structure design has been done from the perspectives of numerical simulation and experimental verification, and a lot of optimum models and stack structure were presented, enabling reinforced mass transport, homogeneous distribution of temperature, reactant concentration, current density, etc. in the cell stack. Combining the advances in materials and optimum cell stack structure, different kinds of zinc-based flow battery cell stacks with high performance have been assembled and reported. For instance, integrating refreshing electrolyte chemistry, a kW-class zinc-iodine flow battery cell stack was assembled and delivered an energy efficiency of ∼80% at 80 mA cm^-2^ (∼53 mAh cm^-2^) for > 300 cycles [16]. The upscaling and performance of alkaline zinc-iron flow battery cell stack ranging from 300 W to 4000 W assembled with hydrocarbon-based cation-exchange membranes were reported and evaluated recently [11], of which a 4000 W cell stack with an areal capacity of 60 mAh cm^-2^ demonstrated an energy efficiency of ∼85% at the current density of 80 mA cm^-2^ (Fig. 2a and c). Since the capacity of a zinc-based flow battery system is determined by the cell stack, not by the volume of the electrolyte, increasing the areal capacity is of utmost importance to reduce the capacity cost of zinc-based flow batteries. Aiming to increase the areal capacity, the distance between membrane and anode as well as the flow field structure in zinc-bromine flow battery cell stack was recently optimized, yielding a 10-kW cell stack that delivers a discharge energy of 31.6 kWh at a high areal capacity of 140 mAh cm^-2^ (40 mA cm^-2^) (Fig. 2b and d) [17]. This cell stack surpasses the limit power rating and areal capacity of many of the existing zinc-bromine cell stacks, including those reported from Redflow (3∼5 kW/maximum 10 kWh energy output [18]).

**Fig. 3:**
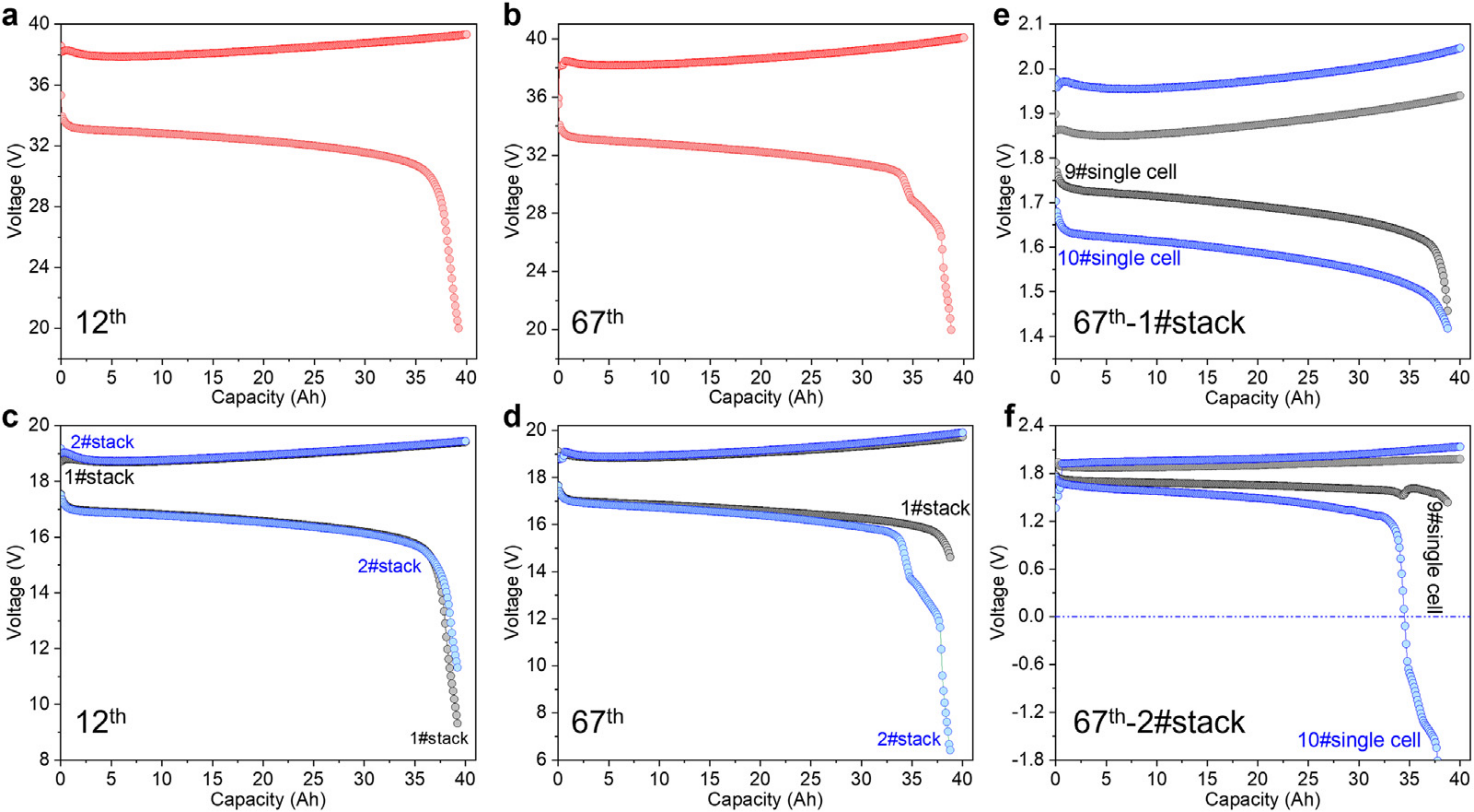
The (a) 12th and (b) 67th charge-discharge curve of two series alkaline zinc-iron flow battery cell stacks (each cell stack was constructed by pressing 10 alkaline zinc-iron single cells together) at the current density of 80 mA cm^-2^. The (c) 12th and (d) 67th charge-discharge curves of each cell stack. The 67th charge-discharge curves of 9#single cell and 10#single cell for (e) 1#stack and (f) 2#stack.

Although many kinds of zinc-based flow battery systems have been reported, few cell stacks have been integrated and their performances, especially the long-term durability performance at high working current density or areal capacity have not been fully evaluated. This is adverse to the practical application of zinc-based flow battery systems. Critically different from the single zinc-based flow battery or the liquid-liquid flow battery cell stack, the zinc-based flow battery cell stack suffers from a relatively low reliability. The higher power normally means a higher working current density or a higher number of single cells. This can easily induce a lower reliability for a cell stack since it is very difficult to ensure voltage consistency for each single cell that results from the heterogeneous mass transfer process in each single cell. Taking the two series alkaline zinc-iron flow battery cell stack as an example, the voltage consistency of the 12th cycle between the cell stacks is very good (Fig. 3a and c). As the cycling proceeds (67th), the two series stack demonstrates an abnormal discharge curve (Fig. 3b), where 2#stack shows two discharge voltage platforms, while the capacity or energy in 1#stack is not fully utilized (Fig. 3d). This can significantly decrease the energy efficiency of the cell stack. Moreover, accumulated capacity in the cell stack can create a vicious circle and ultimately cause the cell stack failure. Thus, challenges remain for the demonstration and practical application of zinc-based flow battery systems prior to adequately evaluating the reliability of the cell stack, especially the reliability of the cell stack working at a high areal capacity.

## 4 Demonstration systems for zinc-based flow batteries

**Fig. 4:**
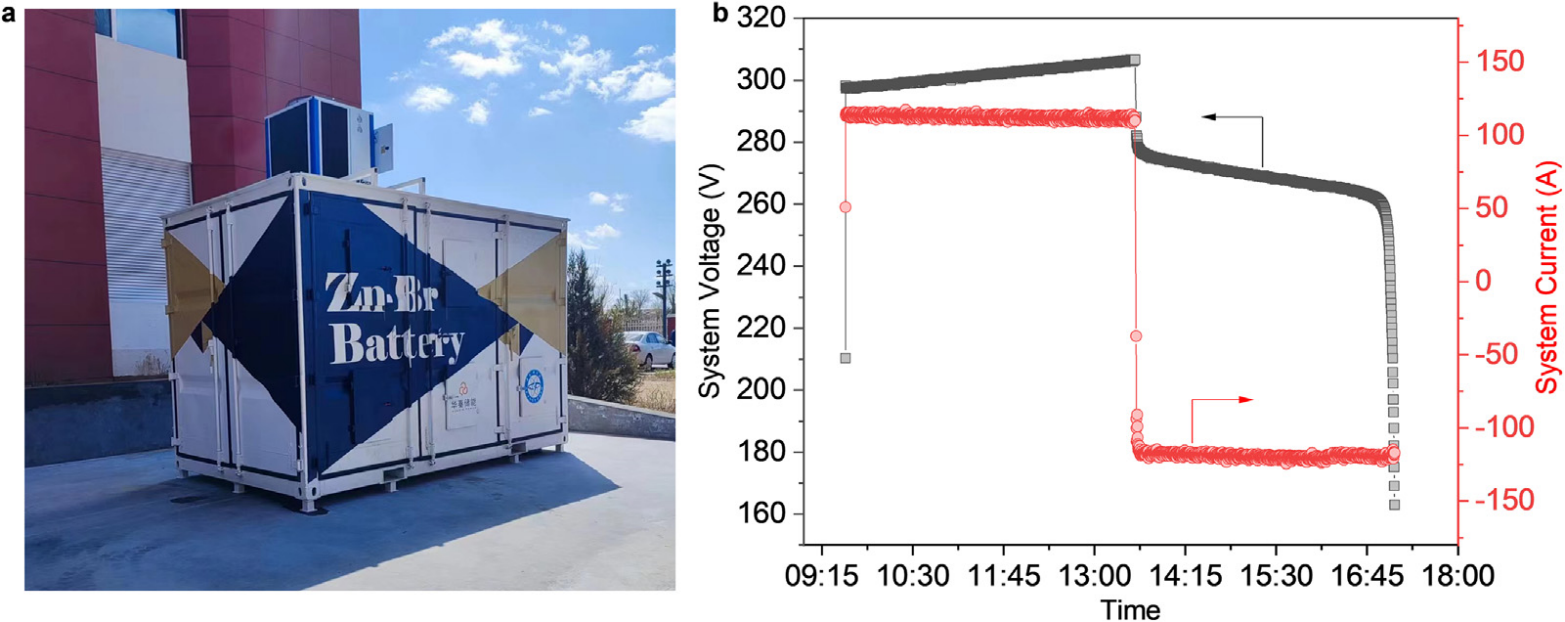
(a) Photograph of 100 kWh zinc-bromine flow battery system. (b) The voltage (current)-time curves of the system.

The history of zinc-based flow batteries is longer than that of the vanadium flow battery but has only a handful of demonstration systems. The currently available demo and application for zinc-based flow batteries are zinc-bromine flow batteries, alkaline zinc-iron flow batteries, and alkaline zinc-nickel flow batteries. Notably, the zinc-bromine flow battery has become one of the most mature technologies among numerous zinc-based flow batteries currently in existence, which holds the most promise for the future. Compared with other redox couples, ZnBr_2_ is highly soluble in the electrolyte, which enables zinc-bromine flow battery a high energy density. The use of ZnBr_2_ for both anolyte and catholyte avoids contamination issues from both sides and makes the recovery of the electrolyte easy. In view of these advantages, many efforts have been devoted to the demonstration and commercialization of zinc-bromine flow batteries. For instance, a lot of zinc-bromine flow battery systems have been installed and implemented based on 3∼5 kW/10 kWh ZBM3 module (Redflow [18]), 25 kW/125 kWh EnergyPod®2 module (Primus Power [19]) and 25 kW/50 kWh ZBB EnerStore® 50V3.1(C) module (EnSync (previously ZBB Energy Corporation since 2015) [20]). In particular, Redflow was awarded US Dept of Energy funds for 34.4 MWh of zinc-bromine flow battery project in September 2023 [21]. In China, the demo and application of zinc-bromine flow battery are pioneered by Dalian Institute of Chemical Physics, Chinese Academy of Sciences (DICP) (who recently implemented a 30∼50 kW/100 kWh system in Yulin (Fig. 4) [22]), Heng'an Energy Storage Technology Co., Ltd (HAES, previously Zbest Power, who has signed a collaboration agreement with Redflow [23]), etc. Although many demonstrations have been installed and progress has been achieved in zinc-bromine flow batteries, challenges remain for their further applications, e.g., the diffusion of bromine redox couple through the membrane and the decreased kinetics of the complexed bromine couple.

In addition to zinc-bromine flow batteries, the demonstrations of alkaline zinc-nickel flow batteries and alkaline zinc-iron flow batteries have also been reported. For instance, Damon E. Turney et al. at the City College of New York reported a 25-kWh alkaline zinc-nickel flow battery [24]. ViZn Energy Systems Inc. has the product of Z20® zinc-iron flow battery that can deliver 48 to 80 kW power with energy of 160 kWh [25]. In 2018, they authorized their technology to Weijing Energy Storage Technology Co., Ltd and installed a 200 kW/600 kWh system in Jiangxi in 2019 [5]. Benefiting from 23 years of experience in flow battery development, DICP also implemented a 10-kW alkaline zinc-iron flow battery system in 2020 [5]. All these demonstrations lay a solid foundation for the development of zinc-based flow battery technologies. Indeed, it is easy to integrate a system, but the challenge is how to realize the stable operation of the system. A zinc-based flow battery system normally has at least two or more cell stacks and is located outdoors, for which a temperature control system is necessary. Different from the assessment methods of a single cell or cell stack at laboratory scale, in which a continuous charging-discharging method at constant current density is normally adopted, a zinc-based flow battery system discharges at a constant power when a user needs it. The charged system will go through a few hours of resting prior to discharge, which will result in capacity decay because of the corrosion and chemical reaction of the metallic zinc in the anode. This in turn increases the maintenance frequency and cost of the system, and even worse, causes system failure. More importantly, the voltage consistency between the cell stacks (Fig. 3d), the voltage consistency for each single cell in a cell stack (Fig. 3e) is more challenging, especially close to the end of discharge of the system. This difference in the output voltage can bring about a reverse charge in the stack (Fig. 3f), which decreases the energy efficiency of the system. When the difference in output voltage is large, irreversible damage to the carbon-based bipolar plate or electrode could occur and cause system failure. This phenomenon is more serious when the power of the cell stack or the system is greater, which makes large-scale demonstrations (> 10 MW) of zinc-based flow batteries very challenging. Given the capacity or energy of a zinc-based flow battery depends on the size of the battery (or stack), zinc-based flow batteries are not suitable for long-duration energy storage applications. Therefore, a good and mature control system with a voltage equalization strategy for single cell stack and cell stacks is very important to enabling the reliability of a zinc-based flow battery system.

## 5 Conclusion

To realize carbon neutrality in the future, different energy storage technologies with different power and discharge duration (energy) requirements remain urgently needed. The past 20 years have witnessed the evolution of materials, cell stacks, and demonstrations & applications for zinc-based flow batteries, showing great potential for distributed energy storage. Although a lot of news (especially in China) on the start-up or contract signing of zinc-bromine flow battery and alkaline zinc-iron flow battery projects has been reported, it still has a long way to go for their practical applications. The laboratory scale battery materials can enable high-power density and high-areal capacity zinc-based flow batteries; however, their upscaling and application in cell stack and demonstration system operation are yet to be demonstrated and fully evaluated. Although many demonstrations have been implemented, the total installed capacity of zinc-based flow batteries is still very low compared to that of vanadium flow batteries, lithium-based batteries, and lead-based batteries, and the data and results for these demonstrations have been rarely reported, making it difficult to evaluate the practicability and progressiveness of the systems. Consequently, the time-tested advanced materials and demonstration systems are necessary and need to be prioritized to accelerate their progress and real-world application. Meanwhile, the optimization of materials, battery or cell stack structure, the ordering of cell stacks, the operational mode of the systems, etc. also deserve more efforts. Most importantly, collaboration between academics and industry is urgently needed to tackle the scientific challenges and accelerate the development of zinc-based flow batteries. Dedicated efforts from both sides are crucial for realizing a high-reliability, high-performance, and low-cost zinc-based flow battery system that meets user demand.

## Declaration of competing interest

The authors declare that they have no conflicts of interest in this work.
